# A Review of Factors Affecting Anthocyanin Bioavailability: Possible Implications for the Inter-Individual Variability

**DOI:** 10.3390/foods9010002

**Published:** 2019-12-18

**Authors:** Merve Eda Eker, Kjersti Aaby, Irena Budic-Leto, Suzana Rimac Brnčić, Sedef Nehir El, Sibel Karakaya, Sebnem Simsek, Claudine Manach, Wieslaw Wiczkowski, Sonia de Pascual-Teresa

**Affiliations:** 1 Department of Metabolism and Nutrition, Institute of Food Science, Technology and Nutrition (ICTAN-CSIC), Jose Antonio Novais 10, 28040 Madrid, Spain; merveedaeker@gmail.com; 2 Department of Food Engineering, Ege University, Izmir 35100, Turkey; sedef.el@ege.edu.tr (S.N.E.); sibel.karakaya@ege.edu.tr (S.K.); sebnem.simsek@ege.edu.tr (S.S.); 3 Nofima, Norwegian Institute of Food, Fisheries and Aquaculture Research, N-1430 Ås, Norway; Kjersti.Aaby@Nofima.no; 4 Institute for Adriatic Crops and Karst Reclamation, Put Duilova 11, 21000 Split, Croatia; Irena.Budic-Leto@krs.hr; 5 Faculty of food Technology and Biotechnology, University of Zagreb, Pierottijeva 6, 10000 Zagreb, Croatia; srimacbrncic@pbf.hr; 6 INRA, Université Clermont-Auvergne, Human Nutrition Unit, CRNH Auvergne, F-63000 Clermont-Ferrand, France; claudine.manach@inra.fr; 7 Institute of Animal Reproduction and Food Research. Polish Academy of Sciences, 10-748 Olsztyn, Poland; w.wiczkowski@pan.olsztyn.pl

**Keywords:** anthocyanin, variability, bioavailability, food processing, metabolism, microbiota

## Abstract

Anthocyanins are dietary bioactive compounds showing a range of beneficial effects against cardiovascular, neurological, and eye conditions. However, there is, as for other bioactive compounds in food, a high inter and intra-individual variation in the response to anthocyanin intake that in many cases leads to contradictory results in human trials. This variability could be caused at two levels, one at the bioavailability level and the other at the effect and mechanisms of action. In this context, we have thoroughly reviewed the scientific literature on anthocyanins variability caused by variation in bioavailability. Based on the literature reviewed, we have concluded that the variability in anthocyanins bioavailability might be produced by the lack of homogeneity introduced at three different levels: food matrix and food processing, enzymes involved in anthocyanin metabolism and transport, and anthocyanin metabolizing gut microbiota. However, it should be noted that the literature on anthocyanins bioavailability considering inter or intra-individual variability is still very scarce, which makes it difficult to reach any firm conclusion on the main metabolizing enzymes or bacteria that would be responsible for the variability in anthocyanin bioavailability.

## 1. Introduction

Anthocyanins receive attention and are studied both because of their impact on food sensory properties, being responsible for the red-blue color of berries, fruits, and certain vegetables-based products, and because of their health-promoting properties [1,2,3,4,5].

Anthocyanins and anthocyanin-rich foods have been shown to display several biological activities which may have positive implications on human health, including anti-inflammatory, anti-diabetic, and antioxidant activities [2,3]. Especially important is their role in preventing cardiovascular health disease through modulation of risk factors such as blood pressure, platelet aggregation, and vascular function [1,5,6]. Furthermore, the intake of anthocyanins seems to have promising effect on cognitive function in humans, with both acute and long-term intakes [4]. Besides, no negative effect of anthocyanin derivatives has been reported, even after ingestion of very high doses [7].

However, the effectiveness of anthocyanins in protecting consumers against chronic diseases depends on numerous factors, which, among others, are influenced by their bioavailability, that is, absorption, metabolism, distribution, and excretion (ADME). Ingested anthocyanins may be efficacious for one individual, but may not trigger the same effect for another. This variability in response to anthocyanins may be due to an inter-individual variation in the absorption and metabolism of these substances or might also be due to a variability on their intrinsic effect to target genes or proteins. An important role in this variability may be ascribed to intrinsic aspects such as genetics, age, sex, and physiological or pathological states. Genetic variation for enzymes involved in the absorption and metabolism of anthocyanins may result in large differences in the expression of a functional enzyme. The mentioned aspects are part of a phenomenon known as inter-individual variability in response to bioactive compounds.

Differences in a person’s gut microbiota and metabolizing enzymes will influence ADME of anthocyanins and consequently, their bioactivity and exerted health effects. It is also important to note that the chemical form of anthocyanins and the food matrix in which these pigments are dispersed in combination with individual physiological variability may be critical factors determining anthocyanin bioavailability.

Consequently, the same dietary intake of anthocyanins can result in different concentration absorbed and different metabolites and consequently, different health effects. In fact, this could be the reason for conflicting or contradictory results of clinical studies on effects of anthocyanins. The aim of this paper is to thoroughly review previous work on anthocyanins bioavailability, in order to assess inter-individual variation in anthocyanins bioaccessibility in humans and to evaluate how it is influenced by food processing, the variability in the enzymes involved, and human gut microbiota composition.

## 2. Methodology

A systematic literature review was undertaken to better understand anthocyanin metabolism and inter-individual variability after anthocyanin intake. Databases (Web of Science and PubMed) were searched for articles written in English and published between January 2000 and August 2018. Keywords used for the bibliographical search were [anthocyanin OR cyanidin OR pelargonidin OR delphinidin OR peonidin OR petunidin OR malvidin] AND [bioavailability OR absorption OR metabolism OR urinary excretion OR plasma OR urine OR pharmacokinetic OR conjugated OR glucuronide OR sulfat* OR sulphat* OR methylat* OR gut OR colon] AND [volunteer OR in vivo OR intervention OR human]. As a result of these searches, we found 1319 papers in the Web of Science and 1426 papers in PubMed databases. Papers found irrelevant for our objective, duplicated papers, or conflicting ones were excluded from the study. After the elimination, we obtained 86 papers that were in accordance with the aim of this work, that is, influence of the type of food processing, the variability in the enzymes involved in anthocyanin metabolism, and human gut microbiota composition on anthocyanin bioavailability. However, only four of these papers dealt with inter-individual variability of anthocyanins bioavailability.

## 3. Anthocyanin Structure and Distribution

**Figure 1:**
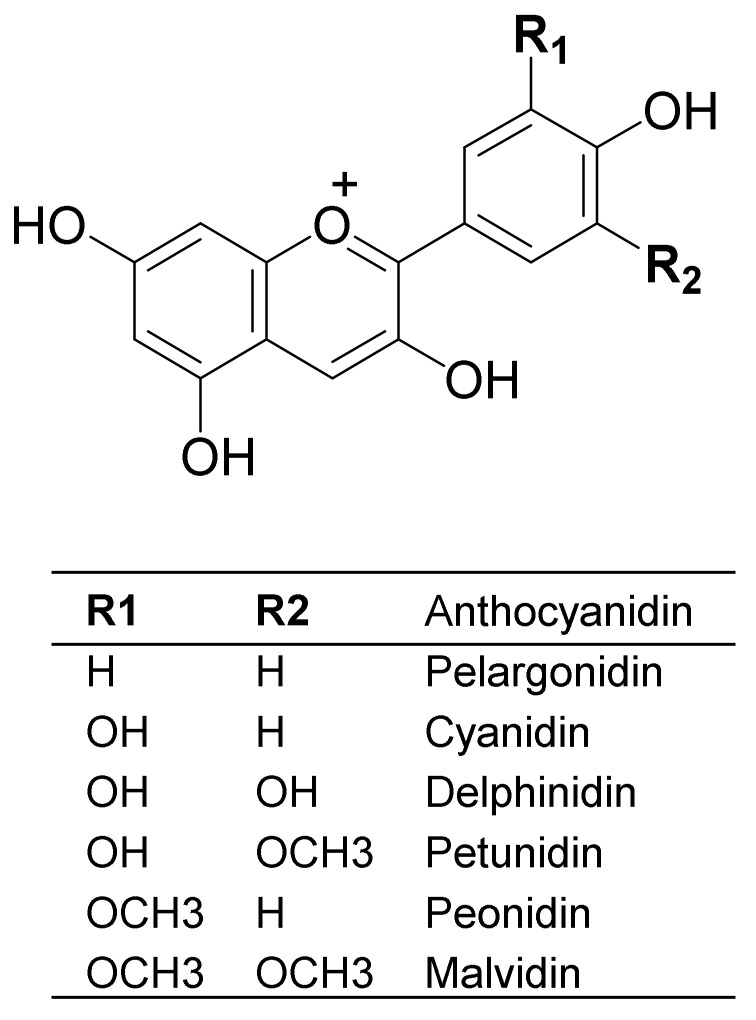
Anthocyanidin structure.

Anthocyanins are water-soluble pigments belonging to the flavonoid group of polyphenols [8,9]. They have glycosidic structure, that is, one or more sugar molecules (glucose, galactose, rhamnose, xylose, or arabinose) bound to the aglycon, the anthocyanidin. The sugar can be acylated with aliphatic acids (malonic, succinic, malic, and acetic acid) or cinnamic acids (p-coumaric, ferulic, and sinapic acid) [8,9]. Anthocyanins differ in (i) the position and number of hydroxyl groups, (ii) the degree of methylation of hydroxyl groups, (iii) the nature and number of sugar molecules attached to the aglycone, and (iv) and aliphatic or aromatic acids attached to the sugar molecule [8]. In nature, more than 600 individual anthocyanins and more than 30 anthocyanidins have been identified [2,9], but only six aglycones are common and widely distributed in foods: pelargonidin, cyanidin, peonidin, delphinidin, petunidin, and malvidin (Figure 1). Most of the anthocyanins in foods are cyanidin derivatives, followed by delphinidin and pelargonidin and the three methylated anthocyanins, peonidin, malvidin, and petunidin [9]. Cyanidin-3-glucoside is the most widely distributed anthocyanin in edible plants and consequently, is the most studied compound [10,11,12].

The main dietary sources of anthocyanins are pigmented fruits and vegetables such as berries, grapes, pomegranates, eggplant, and red cabbage and products derived of these commodities, for example, juices and red wines [7,10]. The daily intake of anthocyanins is estimated to be 12.5 mg per person in the United States [10], however, anthocyanin intake shows a huge variability between individuals due to their content being restricted to a few food items. In this sense, a recent cohort study showed values for anthocyanin intake to be below 9.2 mg/d in the first quintile to more than 23.2 mg/d in the higher quintile [13].

## 4. Effects of Food Processing and Food Matrix

Food processing influences both content and bioaccessibility of anthocyanins. The bioaccessibility of anthocyanins is dependent on the food matrix, on other food components like alcohol and fat, as well as on the structure of the anthocyanin [14]. Acylation increases anthocyanin stability and substantially declines bioavailability [15,16]. Anthocyanidin structure is a determinant of bioavailability, with pelargonidin-based anthocyanins (3′-hydroxyanthocyanins) being more readily absorbed than anthocyanins with more substituents on the B-ring [17]. Structure of the anthocyanidin backbone, the presence or lack of glycosylation and hydroxylation of the basic flavylium structure, and the number, type, and acylation of bounded sugar molecules are linked to plant species and imply characteristic anthocyanins’ patterns. Therefore, the plant species and food matrix are very significant factors that determine the content and bioaccessibility, and thus the bioavailability of anthocyanins. Anthocyanin bioavailability might also be modified by food pH and temperature [14]. Moreover, the presence of other constituents, which were formed and/or added within technological processes, liable to bind, solubilize, or stabilize anthocyanins could modify the bioavailability of the anthocyanins. Food processing, including home food preparation practices, is one of the main factors determining anthocyanins stability and interaction with the food matrix. Several previous studies discuss the effect of plant species, food processing, and food matrix on anthocyanins content. However, knowledge about the impact of the above factors on anthocyanins bioavailability is limited. A summary of studies exploring the effect of thermal and nonthermal processing conditions on anthocyanins content and bioaccessibility and bioavailability are presented in Table 1. In general, thermal processing results in a decrease of anthocyanin contents and an increase of anthocyanins bioaccessibility and bioavailability. Thermal treatments damage cell walls, liberating the cytoplasmic content and making the anthocyanins more accessible for absorption [18,19].

Several in vitro and in vivo studies have reported both positive and negative effects of other co-ingested food components on anthocyanins bioaccessibility [20,21,22,23,24,25,26] (Table 1). These contradictory results may be due to differences in the type of anthocyanin, the degree and type of food processing, including exposure to different pH, temperature, light conditions, and the presence of metal ions or oxygen [27]. A study with hyperlipidemic rabbits reported a higher anthocyanin absorption rate from blackcurrant juice versus an aqueous citric acid solution of purified anthocyanins, suggesting a possible increase in bioavailability resulting from the inclusion of the anthocyanin in a food matrix [28]. On the other hand, in humans, the anthocyanin peak plasma concentration was delayed with co-ingestion of blackcurrant juice with rice cake, whereas no effect was observed for total absorption or excretion of anthocyanins. The results of the study indicated that the rice cake prolonged the stay in the stomach with a slower uptake of anthocyanins from the intestine [28]. A study in humans with consumption of strawberries with or without cream showed no significant difference in plasma and urinary excretion between groups, however, the time required to reach the maximum serum level was delayed in subjects consuming strawberries with cream, suggesting the slowing effect of fat on anthocyanins gastric transit [29]. Additionally, it has been reported that the in vivo antioxidant activity of dark chocolate is reduced by either the effect of milk during intake or the manufacturing process, due to the possible interaction between flavonoids and milk proteins [30]. Additionally, in a human trial, Wiczkowski et al. [25] showed that bioavailability of anthocyanins from fresh red cabbage was higher when compared with fermented red cabbage. The results obtained by Kurilich et al. [20] indicated that the bioavailability of carrot anthocyanins was unaffected by cooking, although cooking increased relative urinary recoveries of nonacylated anthocyanins, but not of acylated anthocyanins.

Also, a few bioavailability studies have investigated the effects of meal composition and other constituents on anthocyanin absorption. Bub et al. [31] suggested that higher glucose content in food products could delay anthocyanins absorption, which could result from competitive action of glucose and anthocyanin on the sodium-dependent glucose cotransporter SGLT1. In another study, Mulleder et al. [32] compared the urinary excretion of anthocyanins when elderberry concentrates were consumed with and without sucrose and showed that urinary recovery of anthocyanins was lower after sucrose consumption. Moreover, the consumption of elderberry juice with sucrose resulted in delaying anthocyanin excretion. A similar phenomenon was found by Walton et al. [33], who in an experiment with pigs showed that that co-consumption of other foods delayed the absorption of anthocyanins from blackcurrant but did not influence the maximum plasma concentration (Cmax) and area under the curve (AUC) values.

Regarding alcohol, results are also controversial. While Bub et al. [31] showed that bioavailability of malvidin-3-glucoside was not significantly different after the consumption of either red wine or dealcoholized red wine in absolute terms, they found that alcohol was able to speed up the absorption (tmax = 50 min for wine and 90 min for dealcoholized red wine). However, Frank et al. [34] did not find that the bioavailability of anthocyanins was significantly affected by alcohol consumption.

McDougall et al. [35] studied the bioavailability of raspberry anthocyanins using an in vitro digestion system, showing that anthocyanins were stable to gastric conditions, whereas they were poorly recovered following pancreatic digestion and that the bioavailability was not affected by joint consumption with different complex food products. Similarly, Sengul et al. [36], using an in vitro gastrointestinal digestion model to study the bioaccessibility of pomegranate anthocyanins, reported that 89% of anthocyanins were stable in gastric conditions, whereas this ratio was just 38% after pancreatic digestion, and only 12% was available in the serum. Among the studied foodstuffs, only meat, soymilk, and cream co-digestion resulted in a loss in serum anthocyanin levels. They concluded that proteins decreased and carbohydrates and fatty acids increased the total anthocyanins content in serum.

Conversely, Ribnicky et al. [37], when investigating the effects of different digestive conditions on the bioaccessibility of blueberry anthocyanins using the Netherlands Organisation for applied scientific research (TNO) gastrointestinal model (TIM-1), concluded that the lipid-rich matrices did not affect anthocyanin bioavailability, whereas protein-rich matrices protected anthocyanins during transit through the upper digestive tract.

Again, in an in vitro gastrointestinal digestion model, Pineda-Vadillo et al. [26] concluded that anthocyanins remain stable and soluble during the oral and gastric steps of digestion, whereas many of them are extensively modified or insolubilized during the intestinal step of digestion. Both thermal degradation and fat and protein interactions decreased anthocyanin bioavailability in this study. Regarding other food components, docosahexaenoic acid (DHA) significantly decreased the bioaccessibility of anthocyanins [38].

## 5. Effect of Enzymatic Variability

After consumption, a very low proportion (<2%) of the original anthocyanins are retrieved in the circulation (plasma/urine) [39]. However, we now know that after ingestion, anthocyanins are subjected to pH-dependent transformations and are hydrolyzed by several enzymes in the small intestine [40]. The released aglycon can enter the epithelial cells by passive diffusion, or a sodium-dependent glucose transporter (SGLT-1) can be involved in the transport of the glycosides (anthocyanins). A portion of the ingested anthocyanin reaches the large intestine, where they are metabolized by the gut microbiota to a range of low-molecular-weight catabolites (phenolic acids and other phenols), which are excreted in the feces or absorbed again. Once absorbed, the anthocyanins and their catabolites undergo phase 2 enzymatic metabolism, first in the wall of the intestine, then are further converted in the liver, that is, the compounds formed are glucuronidated, sulphated, and methylated, catalyzed by UDP-glucuronosyltransferases (UGTs), sulphotransferases (SULTs), and catechol-O-methyltransferases (COMT), respectively. Anthocyanins present the particularity, when compared with other flavonoids, of being absorbed in their native form, as glycosides [41].

Conjugation reactions, particularly sulfation, glucuronidation, and methylation, are known to be involved in the metabolism of phenolic compounds in the human body, generally resulting in stabilization and increased water solubility, and therefore, modifying their distribution and excretion. Sulfation of phenolic compounds is mediated by cytosolic sulfotransferases (SULTs). SULTs catalyze the transfer of a sulfonate group from 3-phosphoadenosine-5-phosphosulfate to a substrate containing a hydroxyl group. The isoforms SULT1A1, SULT1A3/4, SULT1B1, SULT1E1, and SULT2A1 are considered the most relevant in polyphenol metabolism in human adults. Methylation catalyzed by COMT is another metabolic pathway of phenolic compounds [42]. Protocatechuic acid-*O*-sulfate, vanillic acid-4-*O*-sulfate, and ferulic acid-4-*O*-sulfate were plasma metabolites of 13C-labelled cyanidin-3-glucoside [43].

Glucuronidation of phenolic compounds is mediated by the uridine diphosphate (UDP)-glucuronosyltransferases. Glucuronidation is a major metabolic pathway that promotes the elimination of drugs and other potentially toxic exogenous and endogenous compounds, and the existence of inter-individual variability in drug glucuronidation leading to ineffective drug levels or drug toxicity has been reported (Table 2). The individual UGT isoforms expressed in the human liver and ranked from most to least abundant are UGTs 1A1, 1A3, 1A6, 1A9, 2B4, 2B7, 2B10, 2B15, and 2B17. Also, variability in UGT activities has been reported. Possible factors influencing UGT activity variability have been reported as age, sex, genetic polymorphism, and enzyme inducers. Several reviews reported the ontogeny of hepatic drug glucuronidation [44,45]. According to these reviews, prior to, and immediately following, birth, the liver appears to have limited ability to glucuronidate drugs. In initial studies, it was shown that fetal human livers had very low ability to glucuronidate phenolic substrates, which are glucuronidated by UGT1A [46]. Strassburg et al. [47], studied UGT expression in fetal, pediatric (6–24 month), and adult human livers, showing that none of the UGTs were detected in fetal human liver. In contrast, all UGTs expressed in adult liver were determined in pediatrics by 9 months of age. Bolling et al. [48] concluded in a study done in rat liver microsomes from male F344 that the capacity for flavonoid glucuronidation by rat liver microsomes was dependent on age, UGT isoenzymes, and flavonoid structure. Old age appears to have a minimal effect on UGT function.

Several UGTs including UGT2B15 and UGT1A6 have been shown to display higher activities in males than in females. Buckley and Klaassen [49] investigated the tissue- and gender-specific mRNA expression patterns of mouse UGT. They observed that UGT1A1, 1A2, 1A5, and 1A10 and UGT2B1, UGT2B5/37/38 are significantly different between male and female mice in liver and kidney. However, UGT1A7, 1A8, and 1A10, which play a key role in the conjugation and detoxification of numerous phenolic compounds, did not show gender-specific differences. Based on these results we can say that UGT activities depend on tissue, gender, and type of UGT.

Enzyme inducers, including coadministered drugs, smoking, and alcohol, may increase hepatic UGT levels [46]. Hollands et al. [21] investigated urinary excretion of strawberry anthocyanins, which are mainly pelargonidin-3-glucoside. The main metabolite identified in the urine samples was a pelargonidin-monoglucuronide. Although the dose excretion curve was linear for all individuals, there was individual variation in urinary anthocyanin yields, probably due to differences in UGT levels or activity. An effect of diet on UGT1A enzyme activity, as measured by serum bilirubin, has been shown. An increase in UGT1A enzyme activity has been reported for vegetable-containing diets, proving that dietary habits may modulate enzyme activity so that phenolic compounds ADME can be affected [50].

Mallery et al. [52] studied patient-specific differences in local retention and metabolism of freeze-dried black raspberries’ components in oral cavities of 10 healthy individuals and investigated metabolic profiling of the enzymes in tissues obtained from 15 donors. They proposed that as the mouth is contiguous with the more distal components of the gastrointestinal tract, oral cavity enteric recycling is logical and plausible. They reported that oral microflora, salivary enzymes, and surface of oral epithelium contributed to intraoral bioactivation of anthocyanins via β-glucosidase activity. High levels of inter-donor differences in β-glucosidase activities were observed. Phase II enzymes including UGTs, COMT, UDP-glucose-dehydrogenase (UDP-Glu-DH), breast cancer resistance protein (BCRP), and β-glucosidase in 15 tissues were determined. Fourteen out of 15 tissues contained sodium-dependent SGLT1. Another important finding was the detection of protocatechuic acid glucuronide in post-rinse saliva. Inter-donor differences in salivary anthocyanin levels were also apparent. The greatest inter-donor variability was noted at the early (0, 5, and 30 min) time points, and most apparent in cyanidin-3-rutinoside (predominant BRB anthocyanin) and protocatechuic acid (stabilized metabolite). Average inter-donor differences at early time points were 5-fold, with ranges from 3.6- to 10.3-fold.

Riches et al. [53] studied different SULT enzymes (SULT1A1, SULT1A3/4, SULT1B1, SULT1E1, and SULT2A1) which were obtained from human adult’s liver, small intestine, kidney, and lung. They detected the five major SULTs in human liver, small intestine, kidney, and lung cytosol and showed variation between individuals and tissues. There was great variation between individuals in SULT in liver, that is, SULT1A1, SULT1B1, SULT1E1, and SULT2A1. Inter-individual variability of SULTA1 attributed to coding region single nucleotide polymorphism and gene copy number polymorphism. SULT1B1 and SULT1A3 are two of the most dominant SULT in gastrointestinal tract tissues, and then SULT1A1, SULT1E1, and SULT2A1, respectively. In kidney, SULT1A1, SULT1B1, SULT1A3, and SULT2A1, at very low levels, have been detected, but not SULT1E1. Conversely, in lung, SULT1E1 showed the higher levels, followed by SULT1A1, SULT1A3, SULT1B1, and SULT2A1. In conclusion, it was shown that the small intestine has the highest total expression levels of SULT, followed by the liver, kidney, and lung, respectively. Strong variations were shown between individuals and tissues. However, no gender-specific or age-specific variations were shown in the SULT liver expression levels.

COMT is ubiquitously present in mammals and catalyzes the O-methylation of a wide variety of endogenous and exogenous catecholic substrates using *S*-adenosyl-l-methionine as the methyl donor. The human COMT exists in a soluble form (S-COMT) and a membrane-bound form (MB-COMT). Bai et al. [54] showed that when the flavonoid catechin was used as substrate, the formation of 3’-O-methylation products was favored over the 4’-O-methylated products and that MB-COMT had higher binding affinity for 3-*O*-methylation than S-COMT. Two COMT genotypes have been described, GG and AA. It has been shown that individuals having the GG COMT genotype had higher urinary methylated epigallocatechin concentrations after consuming tea than those with AA COMT [55]. Besides these enzymes (SULT, UGT, COMT, etc.), Fernandes et al. [45] indicated that brush border membrane enzymes like lactase phloridzin hydrolase (LPH) may participate in hydrolyzation of anthocyanins and may induce an increase in anthocyanin bioavailability. Németh et al. [51] demonstrated that LPH and cytosolic β-glucosidase can hydrolyze different types of flavonoids glycosides, which suggests a possible hydrolysis of anthocyanins by LPH and cytosolic β-glucosidase. Additionally, they observed significant variability in activity of β-glucosidase isolated from different human small intestine samples. These results may serve as a base to better understand the possible effect of enzymatic variations on the inter-individual variations encountered in anthocyanin’s ADME.

## 6. Effect of the Microbiota on Inter-Individual Variability of Anthocyanin Bioavailability

Among the factors that might affect the inter-individual variation of anthocyanins bioavailability, the microbiota composition has emerged as one of the most important and less well known. In the last fifteen years or so, the number of research papers regarding the microbiota effect on different pathological or physiological conditions has increased in an exponential way. Microbiota catabolism leads to production of new anthocyanin metabolites in the human gut. These metabolites may be absorbed by the colon epithelium and induce modulation of the microbiota composition. The bioavailability of anthocyanins depends on gut microflora and their biotransformation mechanisms. Gut microbiota vary on a person-to-person basis and in this sense, an inter-individual variability in anthocyanin ADME could be expected. In general, it is now assumed that if anthocyanins have a beneficial effect on human health, this effect is mediated by what is called breakdown metabolites, formed by the different bacteria existing in the microbiota. However, when searching in electronic databases (Medline PubMed and Web of Science) for anthocyanins and microbiota, less than 200 articles were initially selected. After removal of duplicates and an initial screening, 43 articles were selected for further reading. After detailed analysis of the full text, 12 articles were rejected, due to lack of relevant outcomes, aspects of the study design, and so forth. Finally, articles published between 2005 and 2018 were incorporated in this review (see Table 3, Table 4 and Table 5). Other articles were not selected because, for example, the polyphenol composition of the dietary sources of anthocyanins was too complex or because anthocyanins did not represent a big proportion with respect to the total polyphenol content (i.e., grape juice or red wine extract), although they suggested a role of the human intestinal microbiota on anthocyanins metabolism [56,57].

After raspberry consumption, a large number of polyphenol metabolites were quantified in human urine by Ludwig et al. [58], including the ones already described and others including methyl-, glycine-, glucuronide-, and sulfate derivatives of phenolic acids and phenylpropanoids. Very interestingly, Ludwig et al. [58] showed that due to the early appearance of 4′-hydroxyhippuric acid and ferulic acid derivatives in plasma, these metabolites may be absorbed, maybe after degradation in the upper gastrointestinal tract, without any microflora intervention on their metabolism.

Another reason for not including some papers in the table is the use of food extracts or food products in which anthocyanins do not represent the main source of polyphenols and from which the formation of metabolites cannot be confirmed. One example is the study of Burgos-Edwards et al. [59], in which a number of metabolites, including 3-coumaroylquinic acid, feruloylquinic acid, quercetin-3-rutinoside, or vitexin, were found after fermentation with human feces of a red Chilean currant. However, these compounds are very unlikely anthocyanin metabolites, and it is difficult to conclude about which polyphenol they are derived from.

Early in vitro studies revealed that bacterial metabolism of anthocyanins leads to the cleavage of glycosidic linkages and breakdown of the anthocyanidin heterocycle. As a result of this breakdown, anthocyanins generate new metabolites via gut microbiota. The type and amount of these metabolites hinge on the structure of food matrix, anthocyanin source, gut composition, and bacterial interaction with anthocyanins [68,69]. After colonic fermentation, different gut metabolites are formed from different anthocyanins. For example, after the gut metabolism of cyanidin derivatives, the metabolites generally formed are protocatechuic acid, vanillic acid and p-coumaric acid, 2,4,6-trihydroxybenzaldehyde, gallic acid, syringic acid and 2,4,6-trihydroxybenzaldehyde, 2,3-dihydroxybenzoic acid, 3-hydroxycinnamic acid, and 3-hydroxyphenylpropionic acid [41,60,75,76]. On the other hand, malvidin derivatives bring in syringic acid, p-coumaric acid, 4-hydroxybenzoic, and homovanillic acid [67,74], and pelargonidin-3-glucoside produces tyrosol, hydroxyphenylpropionic acid, hydroxyphenylacetic acid, and p-hydroxybenzoic acid [66].

Other recent studies have shown that red wine and red wine products, which are rich sources of anthocyanin, produce syringic acid, dihydroxylated benzene, catechol/pyrocatechol, vanillic acid, protocatechuic acid, 3-O-methylgallic acid, and 2,4,6-trihydroxybenzaldehyde after colonic fermentation [67,69,77]. However, Sánchez-Patán et al. [56] did not observe a significant increase in protocatechuic, gallic and vanillic acid levels during fecal fermentation of a similar polyphenol source.

Berries are one of the most studied fruits regarding anthocyanin metabolism. Several studies with human feces and rat feces have shown that protocatechuic, 3-hydroxyphenylpropionic, pyrogallol, 3,4-dihydroxybenzoic acid, and tyrosol are the main metabolites formed after colonic fermentation of berries. These studies prove that mono and di-glycosidic anthocyanins are rapidly catabolized by colonic microbiota [71,76]. In some cases, the production of specific metabolites, that is, O-methylated metabolites from malvidin and petunidin increase their bioactivity [78] (Table 5). On the other hand, glycosylation and acetylation of anthocyanins, while conferring increased stability in the gastrointestinal tract, showed a decreased bioactivity. Some authors have reported hippuric acid as the main anthocyanin metabolite derived from microbial metabolism, however, it is well known that hippuric acid is a rather unspecific metabolite that can be formed after ingestion of other families of polyphenols (i.e., proanthocyanins) [75].

Besides, Cheng et al. [61] studied five intestinal bacteria after incubation with mulberry anthocyanins under anaerobic conditions and identified chlorogenic acid, cryptochlorogenic acid, caffeic acid, and ferulic acid. They concluded that protocatechuic acid, chlorogenic acid, and ferulic acid are microbial metabolites formed by bacterial β-glucosidase action. However, they proposed that the production of chlorogenic acid and cryptochlorogenic acid were derived from chemical degradation of anthocyanins and not from bacterial action. However, it should be noted that chlorogenic and cryptochlorogenic acids might well derive from other compounds present in the extract and not only from anthocyanins.

In general, most authors analyze anthocyanin metabolites in in vitro fermentation or in vivo ingestion assays after 8 h or longer. Faria et al. [84] showed that the highest recovery of anthocyanin metabolites in feces was seen after 24 h of consumption. Czank et al. [43] reported that a remarkable part of cyanidin-3-glucoside metabolites did not reach maximal concentrations in fecal samples until 48 h of interaction. They proposed, in this sense, that a longer fecal sampling period (perhaps ≥72 h) might show better recoveries. However, some of the fermentation studies showing the presence of the aglycon cyanidin or the formation of the glucoside of cyanidin after fermentation of the rutinoside used shorter times (e.g., 2 h in Aura et al. [69]).

**Figure 2:**
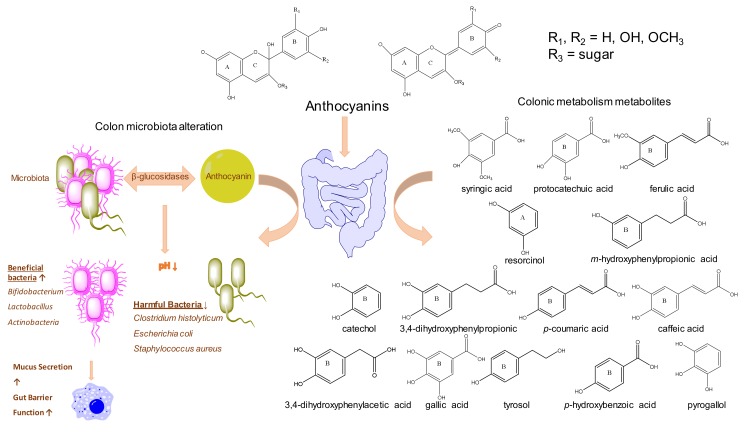
Scheme of Anthocyanins ADME (absorption, metabolism, distribution, and excretion) and structure of the main metabolites.

Several authors have shown that bacterial enzymatic activity affects the catabolism of anthocyanins. Generally, intestinal microbiota has β-*D*-glucosidase, β-*D*-glucuronidase, α-galactosidase, and α-rhamnosidase activity. Gut microbiota breaking of glycosidic linkages and breakdown of anthocyanidin heterocycle are both meditated via bacterial enzymatic activity. This way, anthocyanins are transformed into their more bioavailable forms that, in turn, may regulate the colonic microbiota composition (Figure 2) [61,76,81,84].

Many studies reveal that after the consumption of anthocyanins, beneficial bacteria such as *Bifidobacterium spp*., *Lactobacillus spp*., or *Actinobacteria* populations are increased in the gut microbiota [61,62,63,73,79,82]. These bacteria contribute in different ways to human health as they participate in the metabolism of phenolic compounds, enhance gut barrier function, increase mucus secretion, produce short-chain fatty acids, or regulate lipid metabolism (see Figure 2) [82,84].

A recent in vitro work with bacterial strains demonstrated that after the incubation of black rice anthocyanins, *Bifidobacteria* and *Lactobacillus* increased and pH values decreased [62]. Similarly, Sun et al. [63] reported that after the incubation of peonidin derivatives with different bacterial strains, the pH values decreased and the bacterial growing rate of *Bifidobacterium bifidum*, *Bifidobacterium adolescentis*, *Bifidobacterium infantis,* and *Lactobacillus acidophilus* increased. They concluded that the metabolism of anthocyanins by microbiota bacteria produces the breakdown of β-glucoside bonds and the production of short-chain fatty acids (SCFA) alongside phenolic acids, which triggers the decrease of pH and ensures a suitable media for proliferation of probiotic bacteria.

In another study, after the colonic metabolism of jucara pulp, Guergoletto et al. [70] observed that although *Lactobacillus*/*Enterococcus* spp. did not modify their growing rate, *Bifidobacterium* spp., *Eubacterium rectale*–*Clostridium coccoides* group, and *Bacteroides* spp.–*Prevotella* group did. In parallel, there was an increase in SCFA levels. In a similar way, dietary cherry supplementation promotes SCFA production in gut microbiota and provides an increase in beneficial bacteria (i.e., *Bacteroidaceae*, *Akkermansia*), in this case showing that the increase in SCFA is associated with *Akkermansia* [82]. Similarly, Anhê et al. [80] showed that after consumption of a cranberry extract, there was an increase in *Akkermansia* that was associated with an enhancement of the gut barrier function via mucus secretion stimulation. In the light of all the above, we can conclude that anthocyanins are metabolized by specific bacteria in the gut microbiota and that, in turn, they are able to modify selectively the growing rate of specific bacterial groups [63,65,85]. Moreover, gut microbiota is extremely complex and shows large variability, both intra-individually and inter-individually, depending on the age, body weight, genetics, disease state, and dietary habits. Therefore, we could assume that much of the variability on anthocyanins ADME could be due to changes in microbiota composition [82,85].

Although many studies reported that after anthocyanin colonic fermentation many beneficial bacteria like *Bifidobacterium* spp., *Actinobacteria*, *Bacteroidetes*, *Lactobacillus*/*Enterococcus* spp., *Akkermansia* [62,67,74,80,82,83] increase, Sánchez-Patán et al. [56] failed to show any change for *Bifidobacterium* spp., *Lactobacillus*/*Enterococcus* spp., *Bacteroides*. On the other hand, Overall et al. [78] showed that different profiles of anthocyanins from different sources can have a different impact on the gut microbiota. After colonic fermentation of black berry and black raspberry, *Actinobacterıa* levels remained unchanged, while they proliferated with concord grape, blackcurrant, or blueberry [78]. Besides, they showed that after the consumption of the berries there was a reduction in oxygen tension in the gut lumen that lead to the growth of oxygen-sensitive bacteria.

Anthocyanins can not only enhance beneficial bacteria in gut microbiota, but also reduce some harmful bacteria. Guergoletto et al. [70] and Flores et al. [68] showed that *C. histolyticum* is significantly decreased after colonic fermentation of certain anthocyanins (cyanidin and delphinidin derivatives mainly). However, this effect did not show up when the source of anthocyanin was malvidin-3-glucoside [68]. In addition, Hidalgo et al. [67] indicated that after the colonic fermentation of malvidin-3-glucoside, *C. histolyticum* decreased, although not significantly. Similarly, Sánchez-Patán et al. [56] observed a slight but not significant inhibition of *C. histolyticum*, but did not detect any changes in beneficial bacteria. Trikas et al. [64] reported that anthocyanins of wine and wine by-product extracts inhibited *E. coli* and *S. aureus*. Similarly, Lacombe et al. [83] showed that *Enterococcus* is decreased in feces of rat that were feed with low-bush berry for 6 weeks. These recent studies proved that anthocyanins have the capacity to modulate gut microbiota, bringing out beneficial changes (see Figure 2).

It is difficult to concretely conclude on anthocyanin metabolism and biotransformation by the gut microbiota. Although the scientific literature differs in many ways, such as the source of anthocyanins, food matrix, or in vivo or in vitro model, it can be concluded that anthocyanins and their metabolites generally have a positive impact on microbiota. However, this effect may show variability depending on genetic, physiological, and pathological state. Therefore, the metabolism of anthocyanins by gut microflora and the associated inter-individual variability is an aspect that needs to be better studied in the future.

## 7. Conclusions

There is an evident inter- and intra-individual variability in anthocyanins ADME that will undoubtedly have an effect on anthocyanins efficacy. There are, however, only few studies published so far that describe this variability and even less that aimed to study the role of different of different factors on this inter or intra-individual variability. Nonetheless, looking at the existing bibliography we could assume that there are three main factors that will likely affect anthocyanins ADME, which are food matrix and food processing, enzymatic levels that, in turn, are affected by genetic factors, but also by the diet, age, sex, and so forth, and the microbiota functionality, which again can be modified depending on age, diet, physiological, genetic, and pathological factors.

It is clear from the literature currently available that, even if some of the described metabolites generated through gut bacteria metabolism are quite robust and can probably be used as biomarkers of anthocyanin consumption, more studies are needed in order to establish which species are involved in anthocyanin metabolism in order to clarify the effect of microbiota variability on the inter-individual variation encountered in humans in terms of anthocyanin bioavailability. One can assume that the breakdown products generated from anthocyanins are due either to nonenzymatic degradation or to microbiota activity, but at the moment there are not enough studies in which we can base solid conclusions.

## Figures and Tables

**Table 1:** Effect of technology and processing conditions on bioaccessibility and bioavailability of anthocyanins.

Technology and Processing Conditions	Food	Retention	Effect on Bioaccessibility/Bioavailability	Reference
Microwave cooking (900 W, 12–20 min)	Purple carrots (*Daucus carota*)	↓ (23% total ACs)	↓ 11-14-fold ↓ACs urine and 8-10-fold d↓ ACs in plasma	[20]
Conservation, jam, squeeze	Blackcurrant fruits and products	↓ (0.05–10.3%)	↓ urine (fruit, 0.053%; drink, 0.036)	[21]
Steam-blanching (3 min)	Blueberry puree (*Vaccinium corymbosum*)	↑ ACs *	↑	[22]
Juice processing (milling, mashing, pressing, pasteurization)	Black mulberries Grape/blue berry	↑ (213.8%)	↓	[23,24]
Fermentation (at 18–24 °C, pH 3.80, 14 days)	Red cabbage (Brassica oleracea)	↓ (15%)	↓	[25]
Domestic cooking (45 s–2.5 min, at 250 °C)	ACs enriched food matrices (milkshake, custard dessert, pancake, and omellette)	Very high recovery	≈	[26]

* ACs: anthocyanins. ↑: increase in bioaccesibility/bioavalability of anthocyanins; ↓: decrease in bioaccesibility/bioavalability of anthocyanins; ≈: bioaccesibility/bioavalability of anthocyanins was un-changed.

**Table 2:** Enzymes or enzyme families participating in anthocyanin metabolism that have shown variability at different levels.

Enzyme (or Family)	Isoforms	Metabolites	Variability	Reference
Beta-glucosidases	LPH, cytosolic β-glucosidase	Glucuronides, sulfates	Small intestine	[51]
	GBA1, GBA2, GBA3-1, GBA3-2	Aglycons	Microbiota	[52]
Sulphotransferases (SULT)	SULT1A1, SULT1A3/4, SULT1B1, SULT1E1 and SULT2A1	Sulphates	Tissue	[53]
Uridine diphosphate (UDP)-glucuronosyltransferases (UGT)	UGT1A, UGT2A, UGT2B, UGT3, or UGT8 families	Glucuronates	Age, smoking	[48]
	UGT1A1, UGT1A2, UGT 1A5, UGT1A6	Glucuronates	Sex, tissue	[49]
Catechol-O-methyltransferases (COMTs)	MB-COMT, S-COMT	Methyl substitution	Addictions	[54]
	AA genotype, GG genotype	Methyl substitution	Genetic	[55]

**Table 3:** Microbial metabolites of anthocyanins and bacteria regulated by anthocyanins.

Species	Anthocyanins	Model	Metabolites Found	Bacterial Species	Ef	Reference
Bac	C3G (blackcurrant)	in vitro	3,4-dihydroxybenzoic acid, 2,4,6-trihydroxybenzaldehyde	*Clostridium saccharogumia Eubacterium ramulus*		[60]
Bac	C3G, C3R (mulberry)	in vitro	caffeic acid, ferulic acid, protocatechuic acid, chlorogenic acid, cryptochlorogenic acid,	*Lactobacillus plantarum*, *Streptococcus thermophiles*	↑	[61]
Bac	C3G (black rice)	in vitro	phenyllactic acid, benzoic acid, phenylacetic acid, 2,4,6-trihydroxybenzoic, 4-hydroxyphenylethanol, 4-hydroxybenzoic acid, 4-hydroxyphenylacetic acid, 3-methoxy-4-hydroxybenzoic acid	*Bifidobacteria* *Lactobacilli*	↑ ↑	[62]
Bac	Pn deriv (purple sweet potato)	in vitro		*Bifidobacterium bifidum* *B. adolescentis* *B. infantis* *L. acidophilus* *Staphylococcus aureus* *Salmonella typhimurium*	↑ ↑ ↑ ↑ ↓ ↓	[63]
Bac	Mv deriv (red grape)	in vitro		*E. coli*	↓	[64]
Human	Cy and Pn deriv (purple sweet potato)	anaerobic culture	protocatechuic, phloroglucinol aldehyde, syringic acid, phloroglucinol aldehyde	*Bifidobacterium and Lactobacillus/Enterococcus* *Bacteroides-Prevotella Clostridium histolyticum*	↑ ↓	[65]
Human	P3G (Strawberry) M3G (red grape)	anaerobic culture	*p*-hydroxybenzoic acid tyrosol, Hydroxyphenylacetic. Syringic, vanillic, Hydroxyphenylpropionic acid			[66]
Human	M3G (Red wine)	anaerobic culture	syringic acid	*Bifidobacterium* spp., *Lactobacillus* spp. total number	↑	[67]
Human	C3G, D3R, M3G	anaerobic culture	ferulic, gallic, syringic gallic acid	*C. histolyticum* total number	↓ ↑	[68]
Human	C3G, C3R	anaerobic culture	Protocatechuic acid (3,4-dihydroxybenzoic acid), cyanidin			[69]
Human	C3R, C3G, M3G, P3R, P3G (jucara pulp)	anaerobic culture	gallic acid, syringic acid, benzoic acid	*Bifidobacterium*, *Eubacterium rectale/ Clostridium coccoides*, *Bacteroides/Prevotella group*	↑	[70]
Human	C3G, C3GR (raspberry)	anaerobic culture	3,4-Dihydroxybenzoic acid, tyrosol, catechol, resorcinol, pyrogallol			[71]
Human	M3G, Pn3G, Pt3G (red wine)	anaerobic culture	dihydroxylated benzene, catechol/pyrocatechol, syringic acid	*Bifidobacterium* spp. *Lactobacillus/Enterococcus* *Bacteroides*	NC NC	[56]
Human	C3Ga, C3A (*Arbutus unedo*)	anaerobic culture	3,4-(Dihydroxyphenyl)-acetic acid			[72]
Human	Pn3G, MGa (blueberry)	In vivo		*Bifidobacterium* *Lactobacillus acidophilus*	↑ ↑	[73]
Human	M3G (red wine)	In vivo	syringic acid, p-coumaric acid, 4-hydroxybenzoic, homovanillic acid	*Bifidobacterium*	↑	[74]

C3G: cyanidin-3-glucoside; C3R: cyanidin-3-rutinoside; C3GR: cyanidin-3-glucosyl-rutinoside; Pn: Peonidin; Mv: malvidin; D3R: delphinidin-3-rutinoside; M3G: malvidin-3-glucoside; P3G: pelargodin-3-glucoside; P3R; pelargonidin-3-rutinoside; Cy: cyanidin; Pt3G: petunidin-3-glucoside; C3Ga: cyanidin-3-galactoside; C3A: cyandin-3-arabinoside; Pn3G; peonidin-3-glucoside; MGa: malvidin galactoside; Bac: bacteria; deriv: derivatives; Ef: sense of the regulation, effect; ↑: increase in bacterial growth; ↓: decrease in bacterial groth; NC: no change.

**Table 4:** Microbial metabolites of anthocyanins.

Species	Anthocyanins	Model	Metabolites Found	Reference
Pig	D3G, PT3G, P3G, M3G (red grape)	anaerobic culture	3-*O*-methylgallic acid, syringic acid, 2,4,6-trihydroxybenzaldehyde	[77]
Rat	C3G, C3R, D3R (mulberry)	anaerobic culture	protocatechuic, vanillic, *p*-coumaric acid, 2,4,6-trihydroxybenzaldehyde, gallic acid, syringic acid, 2,4,6-triOHbenzaldehyde	[76]
Rat	Cy deriv (Black raspberry)	In vivo	3-OHphenylpropionic, 3-hydroxybenzoic, 3-OHcinnamic acids	[75]
Rat	C3G	In vivo	protocatechuic acid	[41]

C3G: cyanidin-3-glucoside; D3R: delphinidin-3-rutinoside; M3G: malvidin-3-glucoside; C3R: cyanidin-3-rutinoside Pn: P3G: peonidin-3-glucoside; Cy deriv: cyanidin derivatives.

**Table 5:** Gut bacteria regulated by anthocyanins.

Species	Anthocyanins	Model	Bacterial species	Ef	Reference
Rat	D3G, D3R, C3G, C3R, Pt3R (blackcurrant)	In vivo	*Bacteroides-Prevotella-Porphyromonas group*, *Lactobacillus* spp. *Bifidobacterium* spp., *Clostridium perfringens*	↑ ↓	[79]
Rat	Mv deriv, Pt deriv, D deriv (blueberry) D deriv, Cy deriv (blackcurrant) Mv deriv, Pt deriv, D deriv (red grape) Cy deriv (black raspberry) Cy deriv (black berry)	In vivo	*Actinobacteria*, *Bacteroidetes* *Actinobacteria*, *Bacteroidetes* *Actinobacteria* *Actinobacteria*, *Bacteroidetes* *Actinobacteria*, *Bacteroidetes*	↑ ↑ ↑ NC NC	[78]
Rat	Pn3Ga (cranberry)	In vivo	*Verrucomicrobia Akkermansia*	↑	[80]
Rat	Cy deriv (black raspberry)	In vivo	*Anaerostipes*, *Ruminococcus*, *Akkermansia*, *Coprobacillus Acetivibrio* *Anaerovorax*, *Dorea* *Bifidobacterium*, *Lactococcus* *Anaerotruncus*, *Coprobacillus*, *Desulfovibrio*, *Victivallis*, *Mucispirilum*, *Streptococcus*, *Turicibacter*, *Acetivibrio*	↑ ↓ ↑ ↓ ↑ ↓	[81]
Rat	C3R, C3G, C3G (dark sweet cherry)	In vivo	*Akkermansia*, *Bacteroidaceae*, *Lactobacillus*	↑	[82]
Rat	M3Ga, Pt3G (blueberries)	In vivo	*Proteobacteria Actinobacteria Actinomycetales* *Coriobacteriaceae* *Enterococcus*, *Lactobacillus*	↓ ↑ ↑ ↑ ↓	[83]

C3G: cyanidin-3-glucoside; D3R: delphinidin-3-rutinoside; Pt3R: petunidin-3-rutinoside; M3G: malvidin-3-glucoside; M3Ga: malvidin-3-glacatoside; Pn: peonidin; P3Ga: peonidin-3-galactoside; Mv: malvidin; Pt: petunidin; D: delphinidin; Cy: cyanidin; deriv: derivatives; Ef: sense of the regulation, effect; ↑: increase in bacterial growth; ↓: decrease in bacterial groth; NC: no change.
